# Platelet-Rich Fibrin in Surgical Endodontics: A Report of Two Cases Demonstrating Its Benefits

**DOI:** 10.7759/cureus.78898

**Published:** 2025-02-12

**Authors:** Vaishnavi R Patankar, Ashish K Jain, Rahul D Rao

**Affiliations:** 1 Conservative Dentistry and Endodontics, Bharati Vidyapeeth (Deemed to be University) Dental College and Hospital, Navi Mumbai, IND

**Keywords:** apicoectomy, bone regeneration, periapical granuloma, platelet-rich fibrin, retrograde obturation

## Abstract

Surgical endodontics is the last resort to manage persistent periapical lesions, which do not respond to conventional endodontic treatment. The rationale of periapical surgery is to remove all infected tissues and provide a sealed environment that promotes the healing of periradicular tissues. In order to enhance healing, several biomaterials have been utilized, such as bone grafts, collagen membranes, and platelet concentrates. This case report presents two cases of surgical management of a periapical lesion in failed primary endodontic treatment in the maxillary first premolar and first molar by apicoectomy and the use of platelet-rich fibrin (PRF) for bone regeneration and tissue healing. Both cases were diagnosed as periapical granuloma and showed considerable radiographic bone fill at six and 12 months of follow-up, demonstrating the benefits of PRF in surgical endodontics.

## Introduction

Despite adequate endodontic treatment, few cases develop a persistent periapical infection, which endangers the prognosis of a tooth. Endodontic treatment failures are caused by insufficient chemo-mechanical debridement, bacterial persistence, poor quality obturation, and coronal leakage. Periapical surgery is a final resort to save endodontically involved teeth that cannot be treated conventionally [1]. The purpose of periapical surgery is to remove necrotic and infected tissues and seal all portals of exit from the root canal system with an appropriate retrograde filling material to create an environment conducive to the regeneration of periradicular tissues [2]. The success rate of surgical endodontics is reported to be 88% [3].

The key to tissue regeneration is to initiate a series of synchronized healing events that can result in integrated tissue development [4]. Whether damaged tissues heal by regeneration or repair is determined by the availability of required cell types, as well as the presence or lack of signaling molecules that activate these cells [4]. To stimulate endodontic tissue regeneration and repair, growth factors and host-modulating agents are applied locally to enhance the body's healing potential [5].

Platelet-rich fibrin (PRF) is the second-generation platelet concentrate used in regenerative therapy, which contains platelets, leukocytes, cytokines, circulating stem cells, macrophages, and growth factors such as platelet-derived growth factors (PDGFs), transforming growth factor-1 (TGF-β1), insulin-like growth factors (IGFs), and vascular endothelial growth factor (VEGF), which help to stimulate healing [6]. It has been used in endodontics for pulp regeneration, periapical surgery, endodontic-periodontal lesions, and guided tissue regeneration. This case report describes two cases of surgical management of a persistent periapical lesion in the maxillary first premolar and first molar by apicoectomy and the use of PRF for bone regeneration and tissue healing.

## Case presentation

Case 1

A 26-year-old female patient reported to the Department of Conservative Dentistry & Endodontics with a chief complaint of swelling and pus discharge in the upper left back tooth region for two weeks. She had undergone root canal treatment (RCT) in her upper left back tooth one year ago. The patient had no history of any systemic disease or ongoing medications.

**Figure 1:**
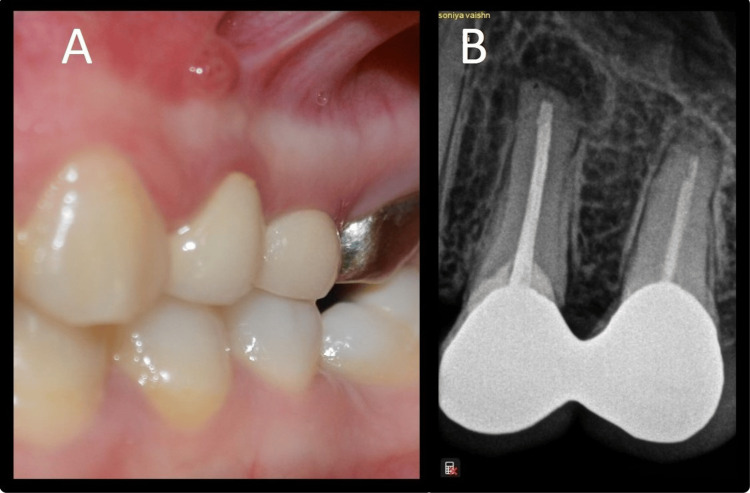
Preoperative images: Case 1 A: Preoperative clinical image, B: Preoperative radiovisiography image

Soft tissue examination revealed swelling present in the buccal vestibule near the apices of the left maxillary first premolar, and on the application of digital pressure, there was yellowish pus discharge (Figures 1A, 1B).

**Figure 2:**
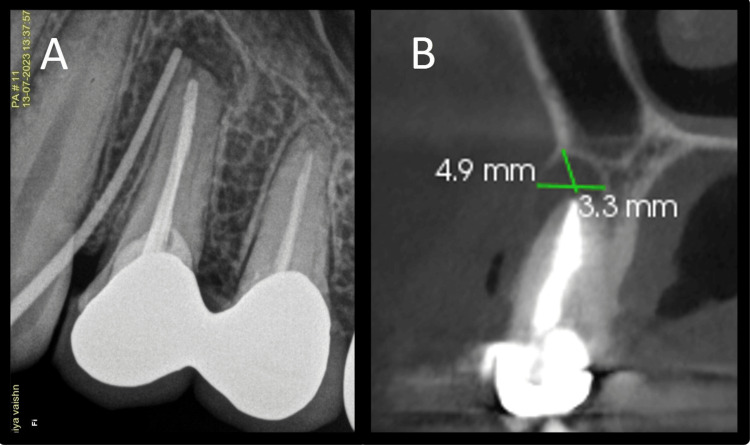
Sinus tract tracing and cone beam computed tomography image A: Sinus tract tracing, B: Cone beam computed tomography coronal view

The intraoral sinus tract was present on the buccal aspect of tooth 24, which, when traced with gutta percha (GP) point, reached the apex of 24 (Figure 2A). Clinical examination showed root canal treated tooth 24 with zirconia single crowns joined together on 24 and 25. There was no tenderness on percussion with 24. Periodontal probing depths were within normal limits. A radiographic examination of the teeth showed dense radio-opaque coronal restoration. There was radio-opaque material in the root canals, suggestive of previous RCT. The quality of the obturation was satisfactory. A thin dense radio-opaque line was seen above the apical plug of GP in the palatal root canal, suggestive of the placement of the fiber post. An ill-defined unilocular periapical radiolucent lesion was seen involving a root apex of 24, along with resorption of the root apex. A diagnosis of a previously treated tooth with chronic apical abscess with 24 was made according to the American Association of Endodontists (AAE, 2013) [7]. As the root canal obturation seemed satisfactory and the prosthesis had a good marginal fit, we did not choose non-surgical retreatment. Additionally, there was a fiber post placed in the palatal canal, removal of which would have resulted in a loss of tooth structure of an already weakened tooth. Thus, surgical treatment was advised to the patient, which included root-end resection, periapical curettage, retro-filling, and placement of PRF in the defect. In order to gauge the size of the lesion and its proximity to the maxillary sinus, a cone-beam computed tomography (CBCT) examination was done (CS 9600 machine (Carestream Health, Rochester, NY) at a field of view (FOV) of 5 x 5 cm under 120 kV and 6.3 mA tube current). The lesion sizes were about 4.9 mm anteroposteriorly and 3.3 mm superoinferiorly (Figure 2B). The patient was advised to do blood tests: complete blood count, hemoglobin count Hb, random blood glucose, hemoglobin A1c (HbA1c), bleeding and clotting time, and activated partial thromboplastin time.

**Figure 3:**
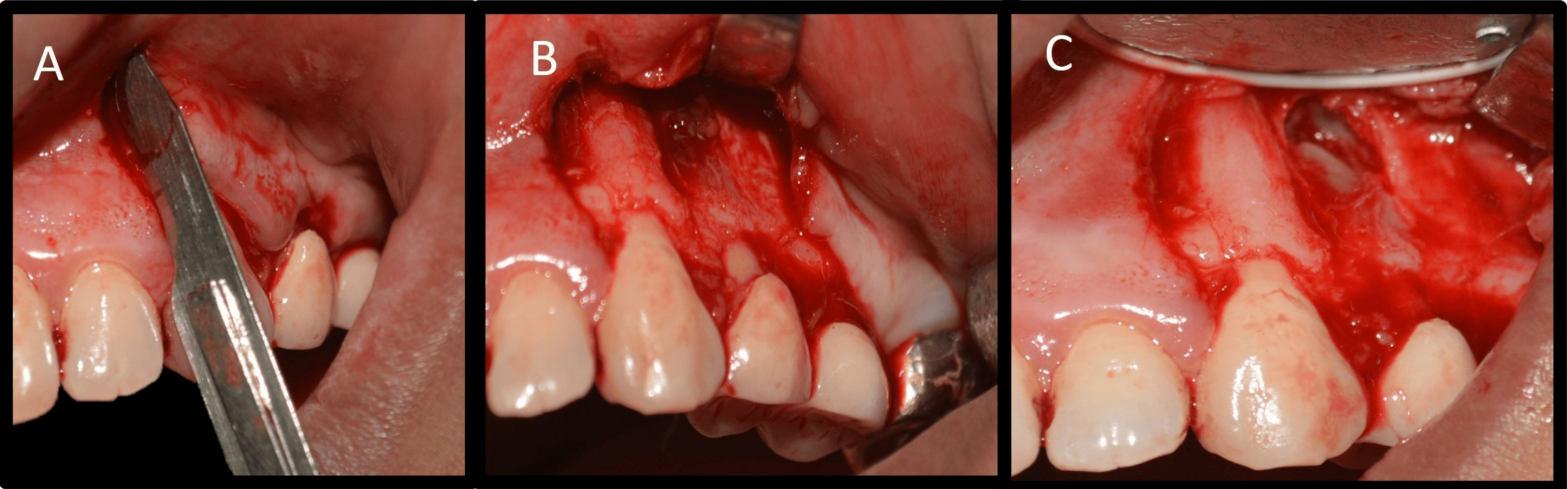
Surgical procedure images A: Incision, B: Flap reflection and localization of the defect, C: Identification of root apices

Informed consent was obtained from the patient. Local infiltration with 2% lignocaine with adrenaline 1:80,000 (Lignox 2% A, Indoco Remedies Ltd., Mumbai, India) was administered. The crevicular incision was made with a 12-number Bard-Parker blade (Swann-Morton, Sheffield, England), followed by two vertical releasing incisions, one mesial to tooth 23, and one distal to tooth 25 with a 15-number Bard-Parker blade (Swann-Morton) (Figure 3A).

**Figure 4:**
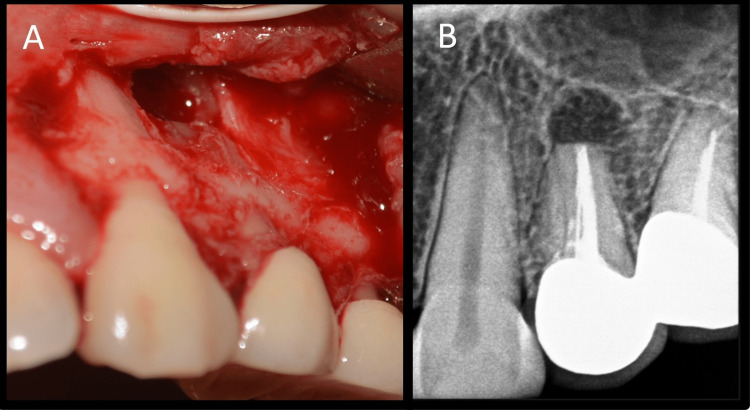
Root end resection A: Root end resection image, B: Root end resection radiovisiography image

As the blood vessels run along the long axis of the teeth, a vertical incision parallel to their course was made to prevent disruption of blood vessels [8]. A rectangular flap was raised. A semilunar flap was not used because it would have provided limited access to the defect and resulted in scarring and wound dehiscence. Full-thickness flap elevation was carried out by periosteal elevator, and the defect was visualized (Figure 3B). The defect was open without any bony cover. The defect was curetted, and all granulation tissue was removed to visualize underlying root apices (Figure 3C). The tissue collected was stored in formalin. Additionally, 3 mm of root end resection was carried out with less than a 10-degree bevel using a straight fissure carbide bur (SS White, Lakewood, NJ) in a micromotor handpiece with continuous irrigation (Figures 4A, 4B).

**Figure 5:**
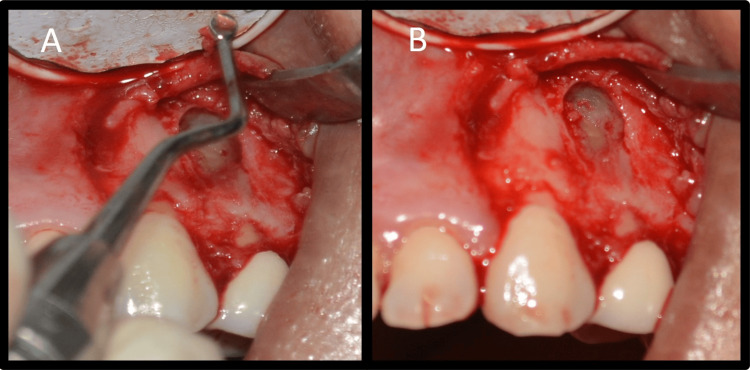
Periapical curettage A: Periapical curettage, B: Healthy underlying bone

Curettage was carried out until underlying healthy bone was seen (Figures 5A, 5B).

**Figure 6:**
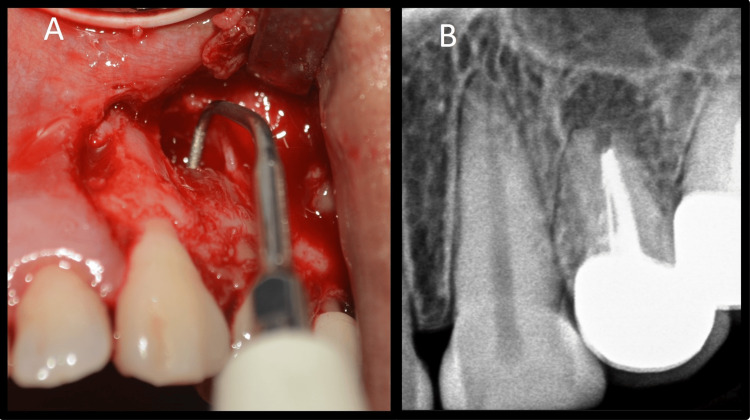
Retrograde preparation A: Root end preparation with a ultrasonic tip, B: Root end preparation radiovisiography image

A class 1 cavity measuring a depth of 2 mm was prepared along the long axis of the tooth with a diamond-coated ultrasonic tip E11D (Guilin Woodpecker Medical Instrument Co., Ltd., Guangxi, China) (Figures 6A, 6B).

**Figure 7:**
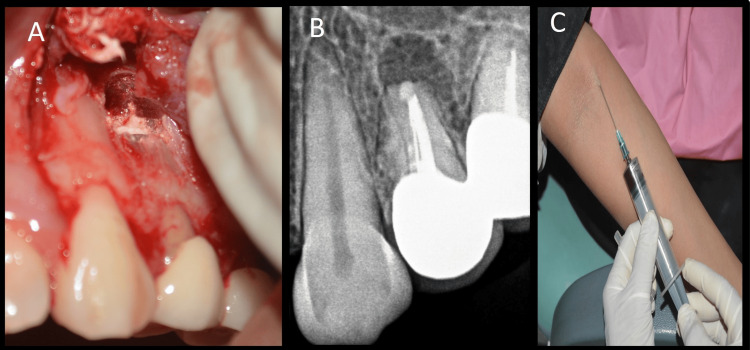
Retrograde obturation & blood collection A: Mineral trioxide aggregate condensation with a plugger, B: Retro-filling radiovisiography image, C: Collection of blood

Adequate hemostasis was achieved with a gauze soaked in 1:1000 adrenaline. After adequate isolation, the root end preparation was filled with mineral trioxide aggregate (MTA; Angelus, Londrina-PR, Brazil) and condensed using pluggers. All excess MTA was removed (Figures 7A, 7B), and 10 mL of venous blood was withdrawn from the patient (Figure 7C).

**Figure 8:**
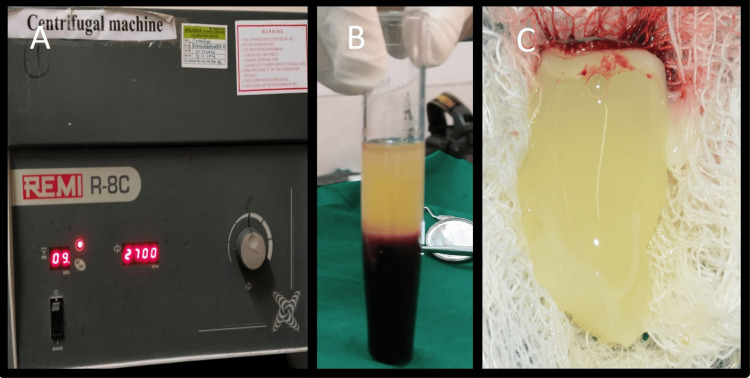
Centrifugation A: Centrifugation to obtain platelet-rich fibrin, B: Test tube showing three distinct layers, C: Fibrin clot

The blood was collected in a sterile test tube without the use of an anticoagulant. The test tube was placed in a centrifugation machine (R8C; Remi Lab World, Mumbai, India) for 12 minutes at a constant speed of 2,700 rpm (Figure 8A) [9].

**Figure 9:**
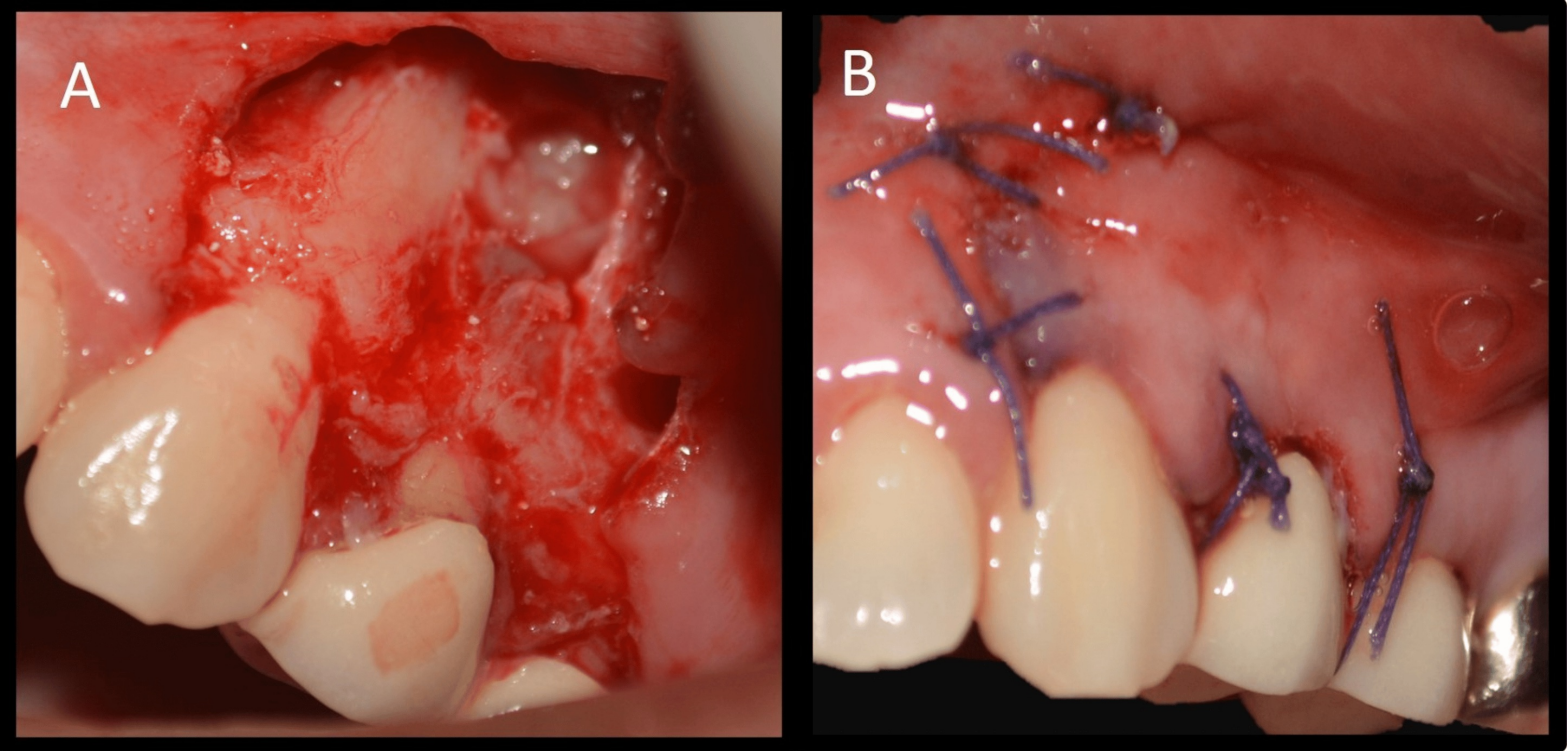
Placement of platelet-rich fibrin in the defect & suturing A: Placement of platelet-rich fibrin in the defect, B: Suturing with interrupted sutures

After centrifugation, three distinct layers are formed: the top layer consists of straw-colored acellular plasma, the middle layer contains the fibrin clot (PRF), and the bottom layer contains red blood cells (RBCs) (Figure 8B). After separating the RBCs and plasma, the fibrin clot (PRF) was collected (Figure 8C). PRF was placed in the bony defect, and the flap was adapted back to its original position and sutured with interrupted polyglactin sutures (4-0 Vicryl; Ethicon, Miami, FL) (Figures 9A, 9B).

The entire procedure was performed under magnification (loupes 3.5x; Zumax Medical Co., Ltd., Suzhou, China).

**Figure 10:**
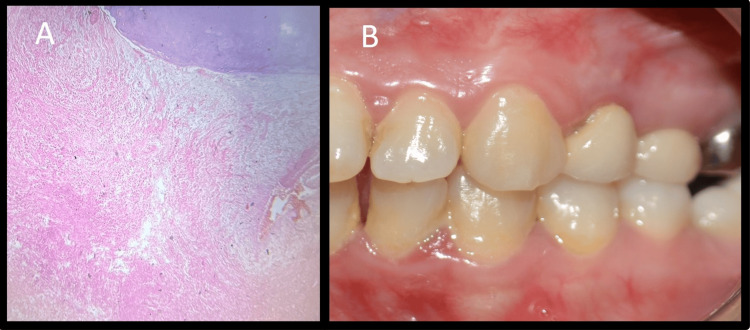
Histopathology & soft tissue healing A: Histopathology revealing periapical granuloma, B: One-week follow-up

Post-surgical instructions were given to the patient. The collected tissue was sent for histopathological examination to the Department of Oral Pathology and Microbiology. The final diagnosis was “periapical granuloma" (Figure 10A).

**Figure 11:**
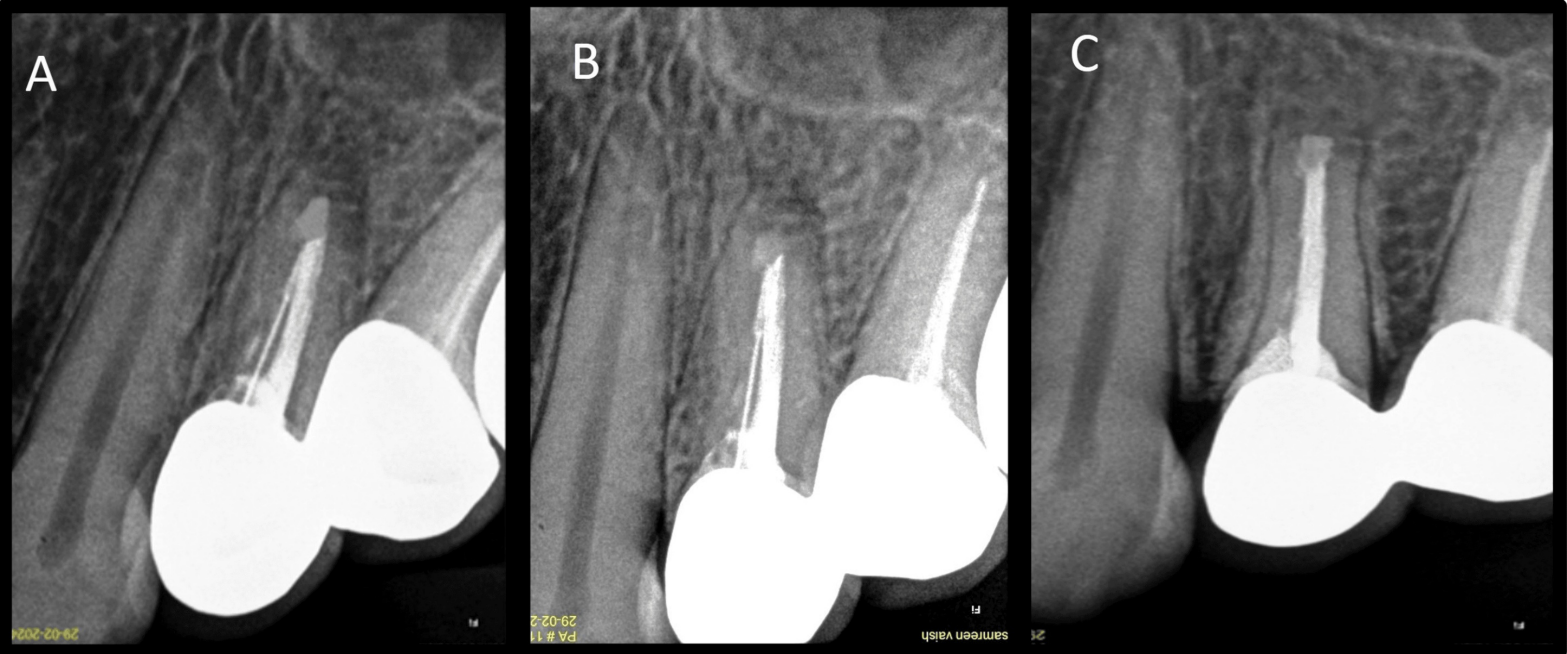
Follow-up images demonstrating radiographic bone fill A: Three-month follow-up, B: Six-month follow-up, C: One-year follow-up

The patient was recalled after one week to check for any signs of infection at the site of surgery and to evaluate if pain was present (Figure 10B). The patient was satisfied following the surgical treatment, as her symptoms had subsided with minimal postoperative discomfort. After seven days, suture removal was done; the patient was recalled for follow-up at three, six, and 12 months; and significant radiographic bone fill was seen at six- and 12-month follow-up (Figures 11A, 11B, 11C).

Postsurgical healing was satisfactory. This case demonstrated complete bone repair according to Molven’s criteria for 2D healing [10].

Case 2

A 32-year-old female patient reported to the Department of Conservative Dentistry and Endodontics with a chief complaint of swelling in the upper right back tooth region for one week. She had undergone re-RCT in her upper right back tooth two months ago. The patient had no history of any systemic disease or ongoing medications.

**Figure 12:**
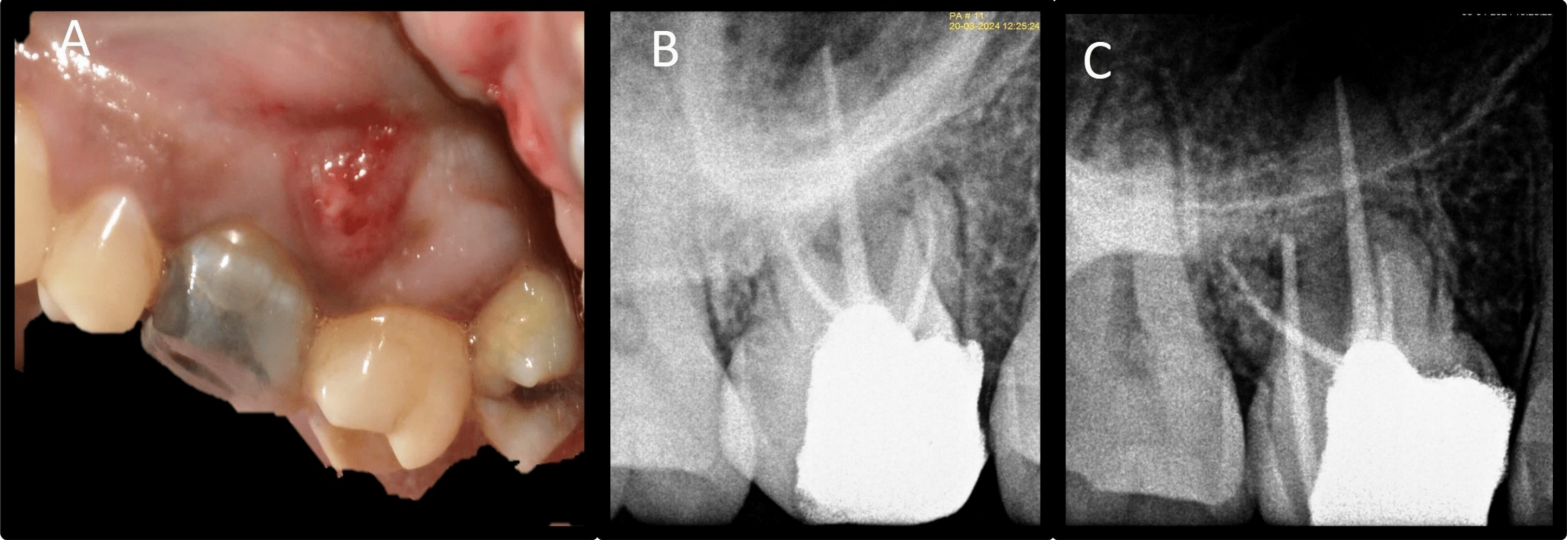
Preoperative images: Case 2 A: Preoperative clinical image, B: Preoperative radiovisiography image, C: Sinus tract tracing,

Soft tissue examination revealed swelling present in the buccal vestibule near the apices of the right maxillary first molar (Figures 12A, 12B).

**Figure 13:**
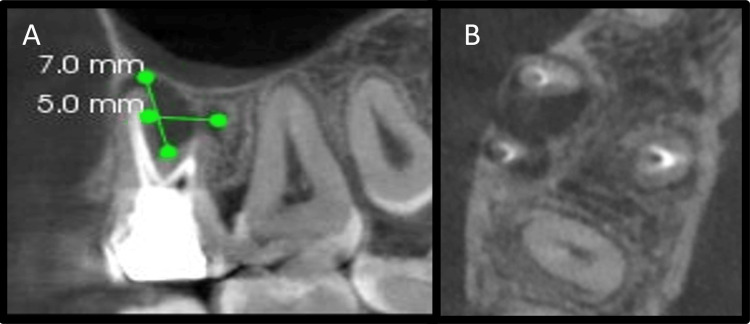
Cone beam computed tomography images A: Cone beam computed tomography sagittal view, B: Cone beam computed tomography axial view

The intraoral sinus tract was present on the buccal aspect of tooth 16, which, when traced with GP point, reached between the apices of mesiobuccal and distobuccal roots of 16 (Figure 12C). Clinical examination showed root canal treated tooth 16 with miracle mix restoration. There was tenderness on percussion with 16. Periodontal probing depths were within normal limits. A radiographic examination of the tooth showed dense radio-opaque coronal restoration. There was radio-opaque material in the root canals suggestive of previous RCT. The quality of the obturation was satisfactory. An ill-defined periradicular radiolucent lesion was seen between the apices of mesiobuccal and distobuccal roots of 16. A diagnosis of the previously treated tooth with chronic apical abscess with 16 was made according to the AAE (2013) [7]. As there was a non-healing lesion, not amenable to non-surgical retreatment, surgical endodontics was the last resort to save the tooth. Surgical treatment was advised to the patient, which included root-end resection, periapical curettage, retro-filling, and placement of PRF in the defect. In order to gauge the size of the lesion and its proximity to the maxillary sinus, a CBCT examination was done (CS 9600 machine (Carestream Health, Rochester, NY) at FOV of 5 x 5 cm under 120 kV and 6.3 mA tube current). The lesion sizes were about 5 mm anteroposteriorly and 7 mm superoinferiorly (Figures 13A, 13B).

The patient was advised to do blood tests: complete blood count, hemoglobin count Hb, random blood glucose, HbA1c, bleeding and clotting time, and activated partial thromboplastin time.

**Figure 14:**
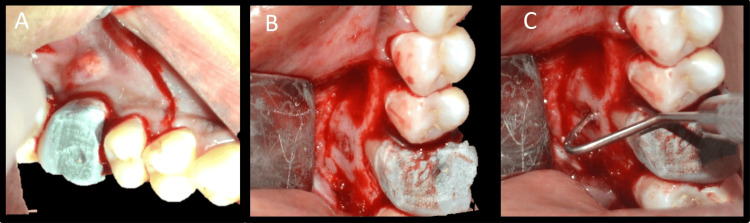
Surgical procedure images A: Incision, B: Flap reflection, C: Localization of the defect

Informed consent was obtained from the patient. Local infiltration with 2% lignocaine with adrenaline 1:80,000 (Lignox 2% A; Indoco Remedies Ltd., Mumbai, India) was administered on the buccal aspect. The crevicular incision was made with a 12-number Bard-Parker blade (Swann-Morton), followed by one vertical releasing incision, mesial to tooth 15 with a 15-number Bard-Parker blade (Swann-Morton) (Figure 14A).

**Figure 15:**
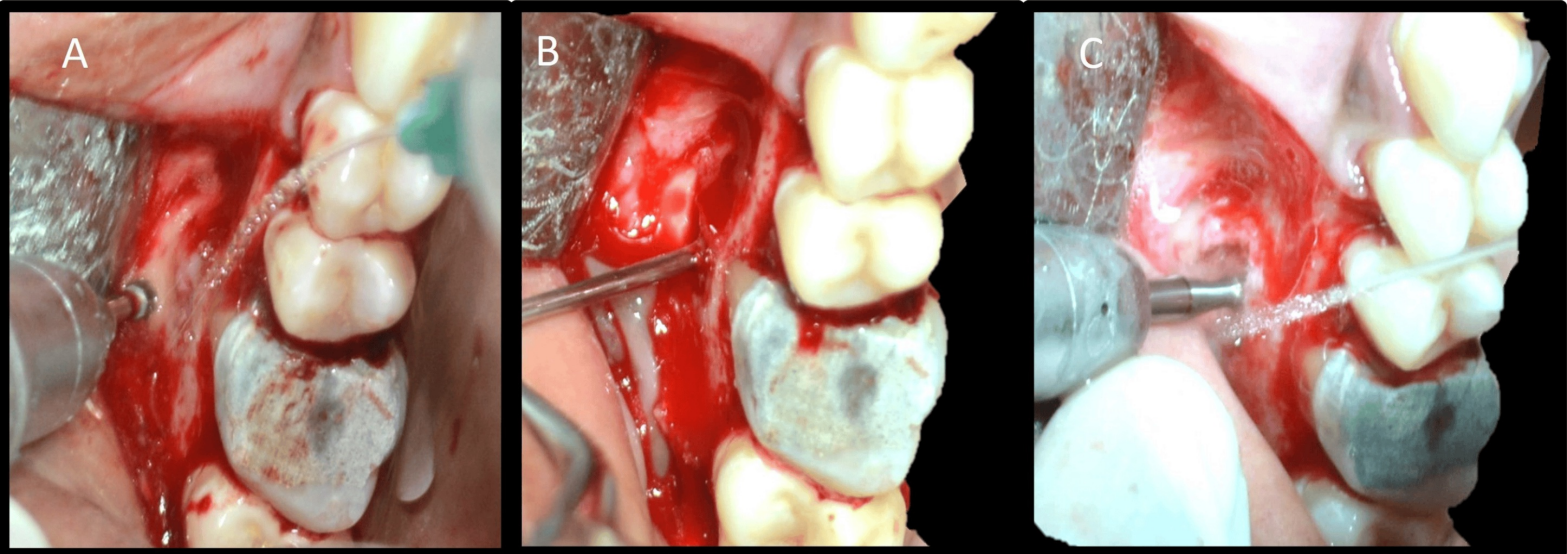
Osteotomy and root end resection A: Osteotomy, B: Periapical curettage, C: Root end resection of the mesiobuccal root

A triangular flap was raised. Flap reflection was carried out by periosteal elevator and the exact position of the defect was localized (Figures 14B, 14C). This case required osteotomy as the buccal cortical plate was intact. CBCT was used to determine the exact position of the root apex, root length, and angulation, along with tactile feedback using a periodontal probe to confirm the position of the apex. Osteotomy was done with a round carbide bur (SS White) in a straight handpiece with adequate irrigation (Figure 15A).

**Figure 16:**
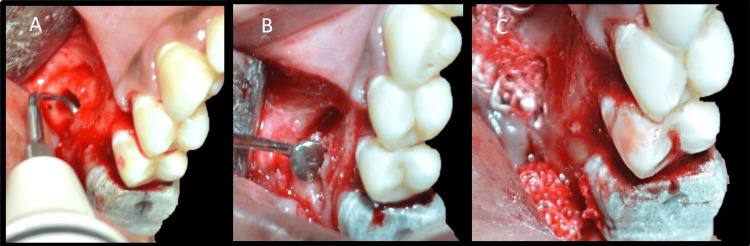
Retrograde preparation and filling A: Retro-preparation with a ultrasonic tip, B: Visualization through a microsurgical round mirror, C: Mineral trioxide aggregate condensation with a plugger

After reaching the defect, it was curetted, and all granulation tissue was removed to visualize underlying root apices (Figure 15B). Additionally, 3 mm of mesiobuccal root end resection was carried out with a minimal bevel using a straight fissure carbide bur (SS White) with continuous irrigation (Figure 15C). The distobuccal root was managed with periapical curettage only. Curettage was carried out until underlying healthy bone was seen. As the lesion was not continuous with the palatal root lesion, it was not treated surgically. A 2-mm-deep class 1 cavity was made along the long axis of the mesiobuccal root using a diamond-coated ultrasonic tip E11D (Woodpecker) (Figure 16A).

**Figure 17:**
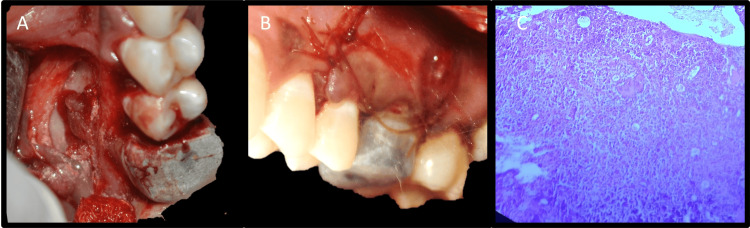
Placement of platelet-rich fibrin A: Preparation and placement of platelet-rich fibrin, B: Suturing, C: Histopathology

A gauze soaked in 1:1000 adrenaline provided adequate hemostasis. Following proper isolation, the root end preparation was filled with MTA (Angelus) and condensed with pluggers (Figures 16B, 16C). All excess MTA was removed, and 10 mL of venous blood was withdrawn from the patient. The blood was obtained in a sterile test tube without the addition of any anticoagulant. The test tube was placed into a centrifuge machine (R8C; Remi Lab World) for 12 minutes at a constant speed of 2,700 rpm [9]. After centrifugation, three separate layers are formed: the top layer is straw-colored acellular plasma, the middle layer includes the fibrin clot (PRF), and the bottom layer comprises red blood cells. After separating the RBCs and plasma, the fibrin clot (PRF) was obtained. PRF was placed in the bony defect, and the flap was adapted back to its original position and sutured with interrupted chromic gut sutures 4-0 (Ethicon) (Figures 17A, 17B).

The entire surgical treatment was done under magnification (loupes 3.5x; Zumax Medical Co., Ltd.).

**Figure 18:**
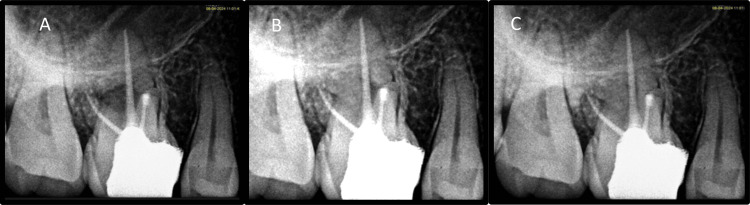
Follow-up images demonstrating radiographic bone fill A: Three-month follow-up, B: Six-month follow-up, C: One-year follow-up

The patient received post-surgical care instructions. The obtained tissue was forwarded to the oral pathology and microbiology section for histopathological evaluation. "Periapical granuloma" was the ultimate diagnosis (Figure 17C). After one week, the patient was recalled to check for signs of infection at the site of surgery and to assess pain, if any. The patient expressed her satisfaction with the surgical procedure because her symptoms were ameliorated with minimum postoperative discomfort. After seven days, sutures were removed, and the patient was recalled for follow-up at three, six, and 12 months. Significant radiographic bone fill was detected at six and 12 months follow-up (Figures 18A, 18B, 18C).

Postsurgical healing was satisfactory. This case showed incomplete bone repair as determined by Molven's criterion for 2D healing [10].

## Discussion

PRF is an innovative step in the platelet gel therapy approach developed by Choukroun et al. in France. The platelets, leukocytes, and cytokines play a significant role in the function of this biomaterial, but the fibrin network that supports them is of great benefit in assembling the key components responsible for PRF's true therapeutic potential [11]. Cytokines are rapidly utilized and destroyed in a healing wound. The balance between cytokines and the fibrin matrix is far more important than any other parameter. The physiologic action of this fibrin matrix is split into four extremely specialized components of healing: angiogenesis, immunological regulation, harnessing circulating stem cells, and wound protection via epithelial cover [12].

There are several benefits of PRF over PRP: it involves an easy and economical process; the use of bovine thrombin and anticoagulants is not needed; favorable healing occurs due to slow polymerization, enhanced cell migration and proliferation, supportive impact on the immune system, and assisting with hemostasis [13]. The success of this method is entirely dependent on the time between blood collection and transfer to the centrifuge, which should be completed in less time. Apart from the benefits, PRF has certain limitations, such as blood handling, limited volume due to its autologous nature, dependence on proper collection of blood and centrifugation methods, rapid resorption rate, and potential difficulties in manipulation [11].

The probable cause of failure of nonsurgical treatment in both cases could be due to insufficient chemo-mechanical debridement, bacterial persistence, or the presence of extra-radicular biofilm and missed second mesiobuccal canal in Case 2. Bone grafts, collagen membranes, and platelet concentrates have been used during endodontic surgeries to manage large periapical lesions. In a clinical trial, the radiographic healing kinetics of PRF and freeze-dried bone allograft (FDBA) were evaluated in patients undergoing apicoectomy. PRF demonstrated significantly faster bone healing than FDBA [14]. The outcome of endodontic surgery depends on the approach used. Endodontic microsurgical approaches outperformed traditional root-end surgery in terms of success rates [15]. In both cases, MTA was used as a retrograde filling material due to its bioactive properties and good sealing ability.

In both cases, clinical and radiographic evaluations at follow-up visits (three, six, and nine months after surgery) demonstrated favorable healing, with no signs of inflammation or discomfort. According to a meta-analysis, individuals treated with PRF experienced significantly less postoperative pain after periapical surgery [16]. The same study assessed periapical bone healing using both CBCT volumetric analysis and qualitative assessment via intraoral periapical radiograph (IOPAR), which yielded superior results with PRF but were not statistically significant [16]. Another study reported that the use of PRF gel in apical surgery yielded promising results by accelerating bone formation after two to three months around periapical surgical defects and minimizing postoperative discomfort [17].

There are several forms of PRF that can be prepared using different centrifugation protocols and tubes. In the above cases, we have used leukocyte-rich PRF (L-PRF). L-PRF membranes have a volume and form easy to incorporate with most surgical procedures, as a filling biomaterial or as a healing membrane [18]. A study found that L-PRF has a favorable antibacterial effect and can release growth factors and matrix proteins for more than seven days [19]. Thus, the use of PRF promotes wound healing and establishes a favorable environment for tissue remodeling.

## Conclusions

This case report highlights the intriguing potential of PRF as a viable biomaterial in periapical surgery, implying its incorporation into dental therapeutic strategies, due to its regenerative qualities and cost-effectiveness. Both cases demonstrated significant bony repair at a six-month follow-up period, suggesting that PRF could accelerate the healing response, emulating the requirements of physiological wound healing. However, clinical studies are required to support this finding.
